# IL-6 Stimulates Intestinal Epithelial Proliferation and Repair after Injury

**DOI:** 10.1371/journal.pone.0114195

**Published:** 2014-12-05

**Authors:** Kristine A. Kuhn, Nicholas A. Manieri, Ta-Chiang Liu, Thaddeus S. Stappenbeck

**Affiliations:** 1 Department of Pathology and Immunology, Washington University School of Medicine, St. Louis, Missouri, United States of America; 2 Department of Medicine, Washington University School of Medicine, St. Louis, Missouri, United States of America; Duke University Medical Center, United States of America

## Abstract

IL-6 is a pleiotropic cytokine often associated with inflammation. Inhibition of this pathway has led to successful treatment of rheumatoid arthritis, but one unforeseen potential complication of anti-IL-6 therapy is bowel perforation. Within the intestine, IL-6 has been shown to prevent epithelial apoptosis during prolonged inflammation. The role of IL-6 in the intestine during an initial inflammatory insult is unknown. Here, we evaluate the role of IL-6 at the onset of an inflammatory injury. Using two murine models of bowel injury – wound by biopsy and bacterial triggered colitis – we demonstrated that IL-6 is induced soon after injury by multiple cell types including intraepithelial lymphocytes. Inhibition of IL-6 resulted in impaired wound healing due to decreased epithelial proliferation. Using intestinal tissue obtained from patients who underwent surgical resection of the colon due to traumatic perforation, we observed cells with detectable IL-6 within the area of perforation and not at distant sites. Our data demonstrate the important role of IL-6 produced in part by intraepithelial lymphocytes at the onset of an inflammatory injury for epithelial proliferation and wound repair.

## Introduction

IL-6 is an inflammatory cytokine that plays an important role in the development of Th17 cells [1]–[3] and contributes to a number of autoimmune diseases, including rheumatoid arthritis [4]. Recently developed humanized monoclonal antibodies that target the soluble IL-6 receptor have become an effective treatment for rheumatoid arthritis leading to improved disease activity scores, decreased acute phase proteins, and decreased joint erosions [5]–[7]. One unforeseen, rare adverse event in these studies was gastrointestinal perforation in patients with a history of diverticulitis [7], [8]. The complication rate of intestinal perforation is currently 1.9 per 1000 patient years. However, the direct attribution of bowel perforation risk to anti-IL6 receptor therapy is challenging in rheumatoid arthritis patient cohorts, as NSAIDs and steroids are often used concomitantly and these drugs increase the risk of bowel perforation [8].

The potential risk of bowel perforation is additionally relevant as IL-6 has also been proposed as a therapeutic target for inflammatory bowel disease (IBD) [9], [10]. Multiple studies have shown that patients with active IBD have highly elevated serum levels of IL-6 and that tissue biopsies contain numerous IL-6-positive mesenchymal cells within the colonic mucosa of inflamed areas [11], [12]. However, in studies using mouse models, there is evidence that IL-6 signaling can be beneficial. IL-6 protects intestinal epithelial cells from apoptosis during toxin-mediated injury with oral dextran sodium sulfate [13], [14] and *C. rodentium* infection [15]. Based on these findings, we hypothesized that IL-6 may have beneficial properties in wound response/repair. As only a small fraction of patients that receive anti-IL-6 signaling therapy have adverse outcomes (i.e. perforation), we surmised that the timing of the therapy with respect to injury was the critical factor that needed to be investigated.

To investigate this question, we utilized two different colonic injury models where the timing of injury induction could be controlled. In both cases, IL-6 was rapidly induced in response to injury induction. We found that this burst of IL-6 expression was required to stimulate intestinal epithelial proliferation, a known component of mucosal wound repair [16]. Importantly, we found that the timing of anti-IL-6 treatment with injury was critical to promote epithelial proliferation in response to damage. In these models, IL-6 was induced early after injury in a population of intraepithelial lymphocytes (IELs) that are in close proximity to intestinal epithelial progenitors. Our findings suggest that treatment with anti-IL-6 therapy can impair the early epithelial proliferative response to injury/inflammation and that this poor response may play a role in increased susceptibility to bowel perforation.

## Results

### IL-6 is produced with induction of intestinal inflammation

**Figure 1:**
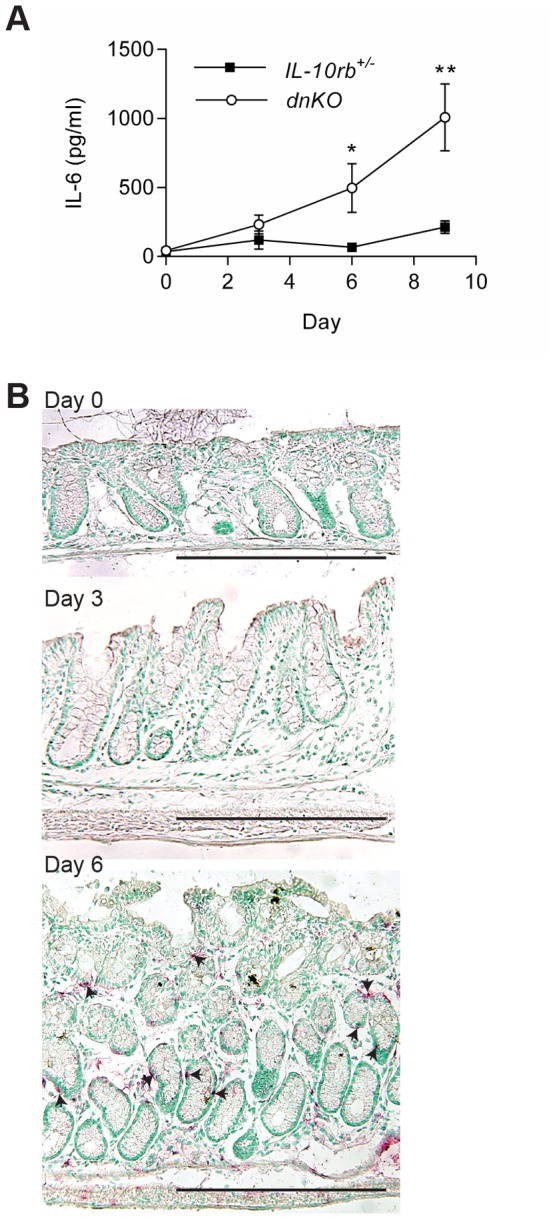
IL-6 was induced with the initiation of colitis in *dnKO* mice. Antibiotic pretreated *dnKO* and *IL-10rb^+/-^* littermate control mice were co-housed with non-antibiotic treated mice to induce colitis in *dnKO* mice. From individual mice, colons and sera were harvested with no co-housing (baseline, day zero) and every three days after co-housing. IL-6 mRNA and protein expression was analyzed by ELISA (A) and *in situ* hybridization (B), respectively. Two experiments were performed with a total of 10–14 mice/group/time point. (A) Plot of the average ± SEM IL-6 protein (pg/ml) in the sera over time for each group of mice. A student's t-test was used to determine statistical significance for each time point; *, p<0.05; **, p<0.0001. (B) Representative images of IL-6 *in situ* hybridization (red staining, arrowheads) are shown for days 0, 3 and 6 post co-housing. Bars = 500 µm.

We first determined the timing of IL-6 expression with respect to the induction of intestinal inflammation. We used *dnKO* mice (transgenic for a dominant negative *Tgfbr2* expressed in T cells and a knockout of the *IL10rb* gene) as this is an established model of triggered colonic inflammation [17]. We have previously shown that after a three week period of antibiotic treatment beginning at weaning, pan-colitis is induced by the introduction of colitigenic bacteria [18]. In this study, we triggered colitis by co-housing antibiotic pre-treated *dnKO* mice with untreated *IL10rb^+/−^* littermates. We first determined IL-6 expression by serum ELISA from samples taken at three day intervals after the initiation of co-housing. We found that day six post co-housing was the first time point where IL-6 was significantly increased in dnKO mice as compared to similar treated *IL10rb^+/−^* littermate controls (Figure 1A). This time point was of interest as it corresponded to the maximal colonization of colitigentic microbes and the induction of colitis [18]. We confirmed the timing of IL-6 induction by *in situ* hybridization of colonic sections taken at days zero, three and six post co-housing. This analysis also showed that IL-6 was first detected at day six post co-housing (Figure 1B). Interestingly, many of the IL-6 positive cells were closely associated with intestinal crypts.

### The effects of IL-6 on the intestinal epithelium are required early after injury

**Figure 2:**
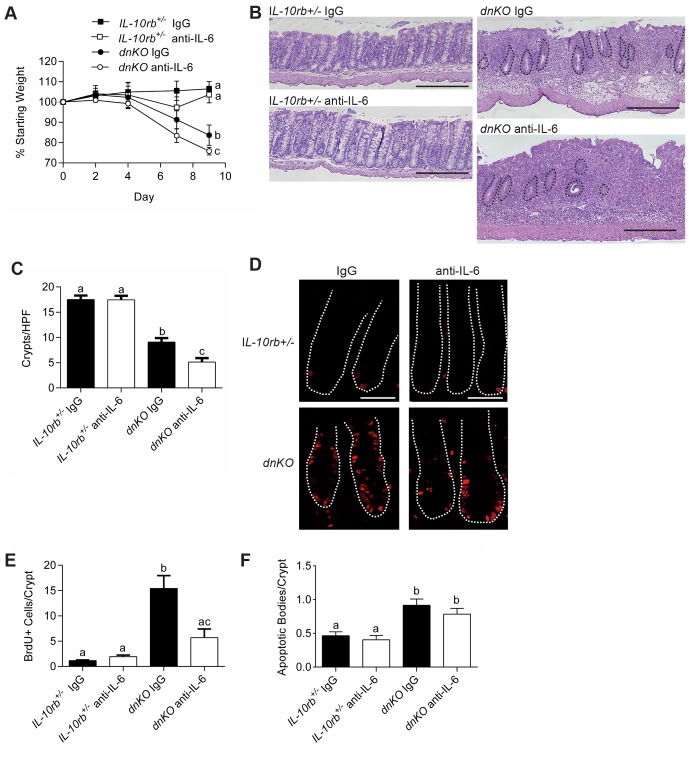
Inhibition of IL-6 resulted in more severe colitis and inhibition of intestinal epithelial proliferation. Colitis was induced in *dnKO* and *IL-10rb^+/-^* littermate controls by co-housing. On day zero and three times weekly, mice were injected intraperitoneally with 500 µg of either anti-IL-6 mAb or control IgG mAb. Two independent experiments were performed with 8-9 mice/group. (A) Plot of the average percent of starting weight ± SEM shown for indicated groups of mice. Mice were weighed every three days. (B) Representative H+E stained sections of descending colons at day 9 post co-housing. Bars = 500 µm. Black dotted lines outline remaining crypts in the *dnKO* anti-IL-6 mAb treated mouse histology. (C) Graph of the average number of descending colonic crypts per high-powered field ± SEM. (D) At day 9 post-co-housing, mice were injected with BrdU one hour before sacrifice. Representative colonic sections stained with mAb to BrdU and detected with fluorescently conjugated antibodies were shown. The white dotted lines delineate crypts. Bars = 100 µm. (E) Graph of the average number ± SEM of BrdU positive cells per crypt. (F) Graph of the average ± SEM number of apoptotic bodies/crypt. One-way analysis of variance: (A) F = 3.5, *P*<0.05 (for day 9 weights); (C) F = 57.36, *P*<0.0001; (E) F = 17.92, *P*<0.0001; (F) F = 10.87, *P*<0.0001. Means with different letters are significantly different by Bonferroni's multiple comparison test.

We next tested the functional role of IL-6 during the induction of colitis in *dnKO* mice. We inhibited IL-6 function by injection of a previously characterized neutralizing monoclonal antibody (mAb) directed against IL-6 [19]. Treatment of *dnKO* mice with anti-IL-6 mAb beginning at the onset of co-housing led to significantly greater weight loss over time as compared to *dnKO* mice injected with a control IgG mAb (Figure 2A). Histologic analysis of the colons in these experiments showed that at day nine post induction, *dnKO* mice treated with anti-IL-6 mAb showed greater evidence of mucosal damage. Notably, we observed increased crypt drop out in the anti-IL-6 *dnKO* group as compared to IgG injected *dnKO* mice (Figure 2B, C). The increased crypt loss suggested a defect in crypt regeneration that might be linked to diminished epithelial proliferation. We tested this idea by analysis of BrdU incorporation into colonic epithelial cells in *dnKO* mice injected with either IL-6 neutralizing or control mAbs. *DnKO* mice treated with anti-IL-6 mAb showed significantly less epithelial proliferation than controls (Figure 2D, E). The effects of anti-IL-6 on crypts and epithelial proliferation were specific to *dnKO* mice as control littermate mice similarly treated did not show any effects from administration of anti-IL-6 mAb.

As IL-6 has been proposed to play a role in cell survival in other damage and infection models [14], [15], we evaluated the effects of anti-IL-6 mAb on epithelial cell death in these experiments. We found that apoptotic cells were not increased within the epithelium of triggered *dnKO* mice with administration of anti-IL-6 mAb as compared to control mAb (Figure 2F), suggesting that in the absence of IL-6, there is cell cycle arrest rather than increased cell death. IL-6 depletion in these experiments was effective, as serum IL-6 levels in mice treated with anti-IL-6 mAb were undetectable (Figure S2). Taken together, these results show that IL-6 function was required for stimulating epithelial proliferation during intestinal inflammation.

### IL-6 is necessary for efficient stimulation of epithelial proliferation after injury

As it appeared that IL-6 was induced at the onset of intestinal inflammation, we next evaluated the induction of IL-6 using a model of intestinal injury with precise timing. Our goal was to more exactly define the timing of the induction of IL-6 with respect to stimulation of epithelial proliferation. For this purpose, we used a model of endoscopic-guided biopsy to produce focal areas (∼1 mm^2^) of acute injury to the colonic mucosa [20], [21]. In WT mice, such biopsy wounds typically heal within two weeks through multiple defined steps. After an initial phase (typically one day) where the wound is capped by wound associated epithelial cells, a second phase of repair is initiated that is driven by an expansion of intestinal epithelial progenitors. This latter phase is important to generate new intestinal stem cells that will populate new crypts that from within the wound bed [16].

**Figure 3:**
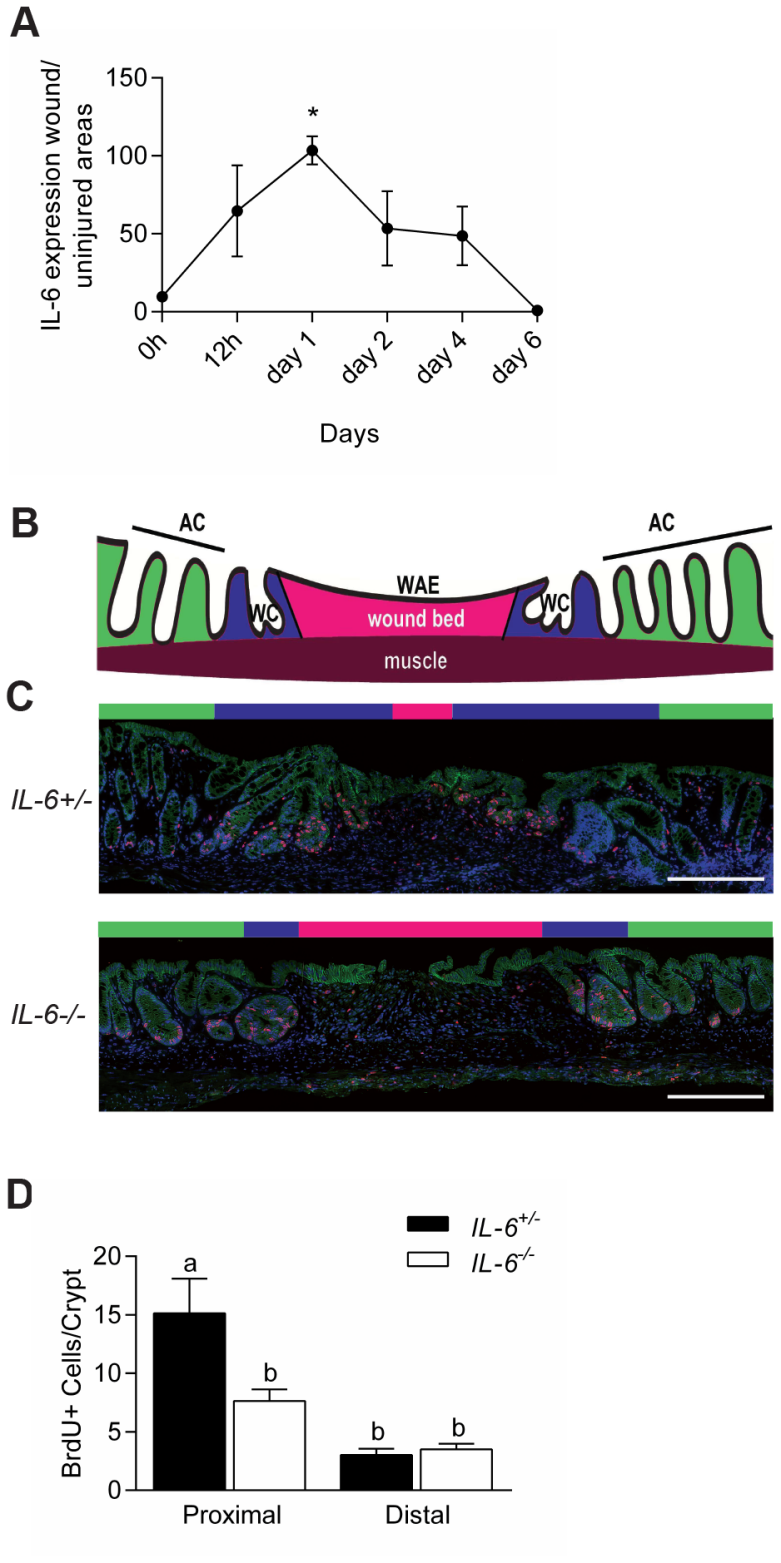
IL-6 promoted intestinal epithelial proliferation in wound biopsy model. (A) WT mice were biopsy injured in the distal colon. Plot of the relative levels of IL-6 mRNA expression in the wound bed (relative to uninjured tissue) for various times after injury. N = 2–3 WT mice with a total of 4-6 wounds/time point. Data were shown as average ± SEM. One-way analysis of variance: F = 5.68, *P*<0.01 (B) Cartoon depicting the microanatomy of a wound at day six post-biopsy; AC  =  adjacent crypts (green area); WC  =  wound channels (blue area); WAE  =  wound-associated epithelium overlying the wound bed (pink area). (C) Colonic sections of wounds from *IL-6^+/−^* and *IL-6^-/-^* mice at day six post-injury stained with mAb to BrdU (labels S-phase cells, red), mAb to β-catenin (labels epithelium, green), and bis-benzimide (nuclei, blue). Bars = 500 µm. (D) Quantification of the number of BrdU positive cells/wound adjacent crypts. Data were graphed as average ± SEM. One way analysis of variance: F = 10.5, p<0.0001. Means with different letters are significantly different by Bonferroni's multiple comparison test.

We first measured IL-6 mRNA levels within excised wound beds at specific times after injury and compared them to uninjured tissue by qRT-PCR. IL-6 mRNA expression was increased within the wound bed by 12 hours post-injury and peaked at 24 hours post-injury. IL-6 mRNA expression levels declined over the next six days post-injury to baseline levels (Figure 3A). Thus, peak levels of IL-6 expression occurred immediately preceding the phase of epithelial progenitor expansion through proliferation.

We then tested the role of IL-6 in epithelial proliferation by comparing biopsy injured *IL-6^-/-^* mice versus *IL-6^+/−^* littermate controls. Specifically, at day six post-injury, *IL-6^-/-^* mice showed diminished epithelial proliferation as compared to control mice in adjacent crypts and wound channels as determined by Ki67 localization (Figure S1) and BrdU incorporation (Figure 3B, C). Thus, IL-6 is important for stimulating epithelial proliferation in response to multiple types of colonic mucosal injury.

### IL-6 induced early after injury is required to promote intestinal epithelial proliferation

**Figure 4:**
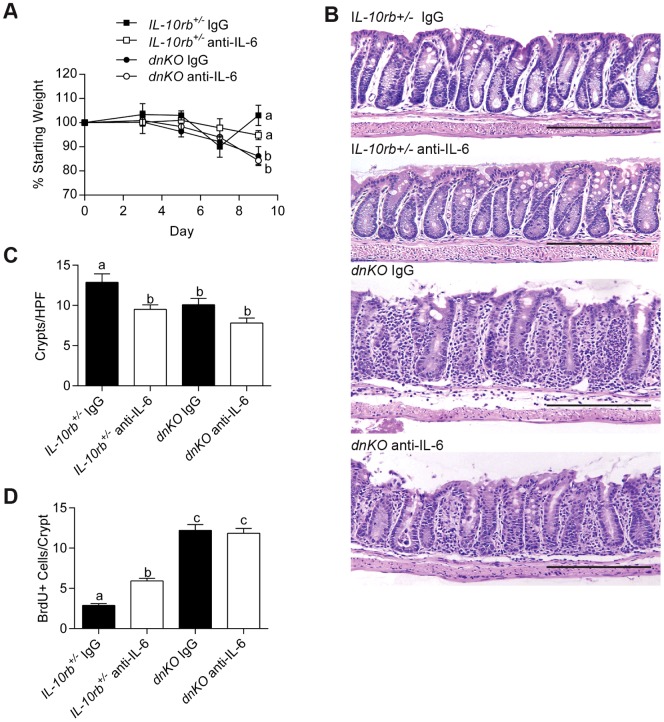
IL-6 inhibition after colitis induction did not inhibit epithelial proliferation but did not improve disease. Three days after induction of colitis in *dnKO* and *IL-10rb^+/-^* littermate controls, 500 µg anti-IL-6 antibody or control IgG was administered to mice three times weekly. Mice were sacrificed 9 days after induction of colitis. 6–8 mice per group in two independent experiments were analyzed. (A) Plot of the average percent of starting weight ± SEM shown for indicated groups of mice. Mice were weighed every three days. (B) Representative H+E-stained sections of descending colons from each group of mice. Bars = 500 µm. (C) Graph of the mean number of crypts/high-powered field ± SEM of the descending colon per animal. (D) Graph of the average number of BrdU+ cells/crypt ± SEM. One-way analysis of variance: (A) F = 4.88, *P* = 0.016; (C) F = 21.14, *P*<0.0001; and (D) F = 68.24, *P*<0.0001. Means with different letters are significantly different by Bonferroni's multiple comparison test.

IL-6 is generated in *dnKO* mice soon after colitis induction. To functionally test if IL-6 was required only at the onset of colitis, we altered the timing of the first administration of anti-IL-6 mAb in triggered *dnKO* mice. We found that a delay of three days in the introduction of anti-IL-6 mAb with respect to the onset of co-housing abrogated the effects on the intestinal epithelium of IL-6 inhibition when the anti-IL6 mAb is introduced at the same time as co-housing. Weight loss in *dnKO* mice treated with anti-IL-6 mAb was not significantly different from *dnKO* mice treated with control IgG (Figure 4A). Analysis of colonic sections showed a similar appearance when comparing *dnKO* mice treated with anti-IL-6 mAb or control IgG. We also found that crypt number was similar between these groups (Figure 4B, C). In addition, BrdU incorporation was not different between *dnKO* mice treated with anti-IL-6 or control antibody (Figure 4D), demonstrating that the later inhibition of IL-6 did not impair proliferation of epithelial stem cells in crypts. Therefore, IL-6 is crucial at the onset of an injury for epithelial proliferation, but at later stages of intestinal healing, IL-6 likely contributes to other processes.

### IL-6 expression is induced in intraepithelial lymphocytes in injured mice

**Figure 5:**
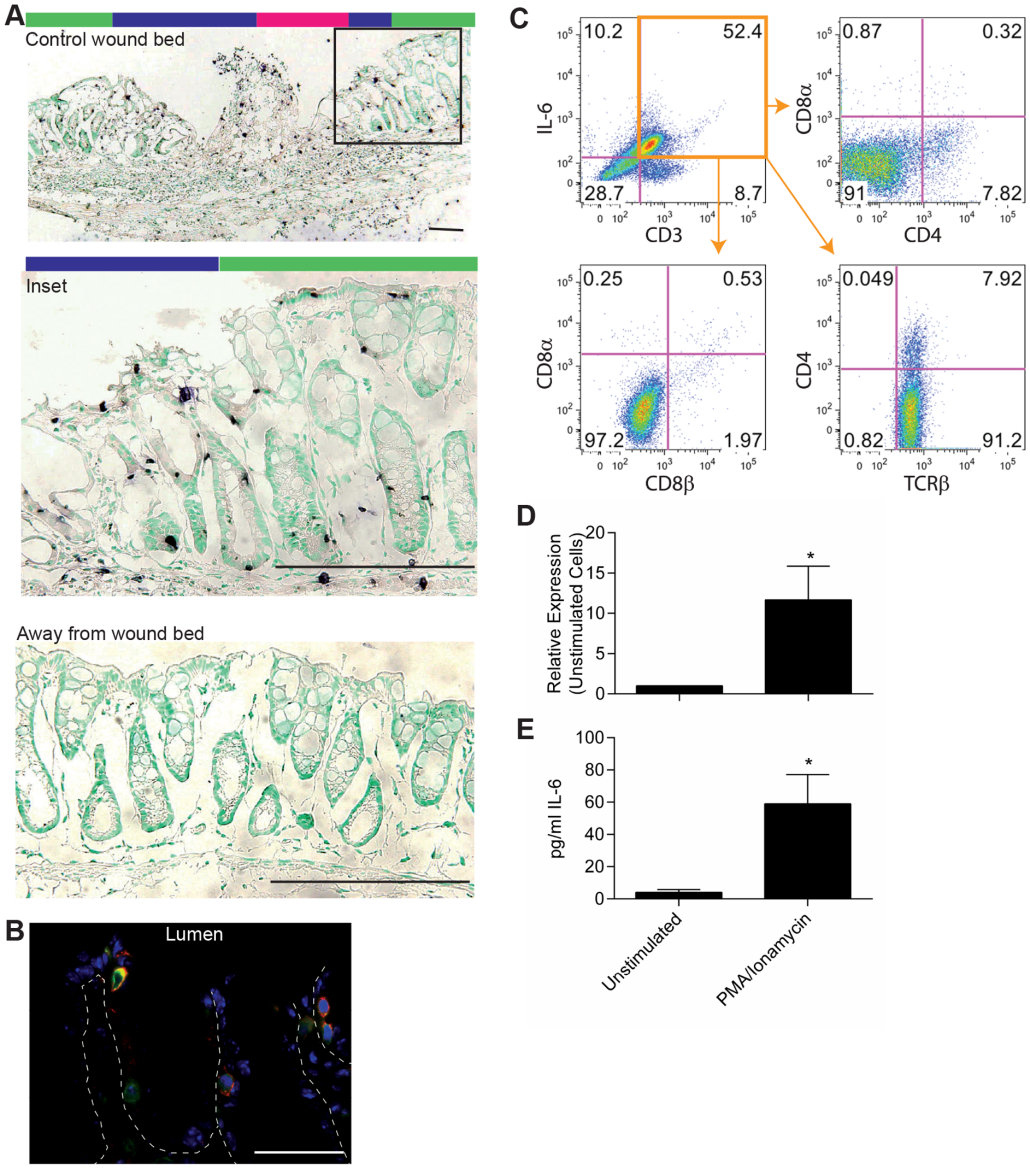
Intraepithelial lymphocytes were a source of IL-6 early after injury. (A) Biopsy of the colon mucosa was performed in WT mice to create small wounds. IL-6 expression in the wound bed and adjacent tissue was evaluated by *in situ* hybridization one day after biopsy. Representative images were shown. Bars = 200 µm. Colored bars above wound images indicate areas of the wound bed as depicted in Figure 4B. (B) Co-localization by immunofluorescence was performed for IL-6 (red), CD3ε (green), and bis-benzimide (blue) on colon tissue from *dnKO* mice at day 6 after co-housing. Representative staining was shown at 63X. Bar = 200 µm. (C) Epithelial cells were harvested from *dnKO* mice on day 6 after co-housing, stained for T cell markers and IL-6, and assessed by flow cytometry. Representative dot plots were shown. (D, E) CD3+ CD4- CD8- IELs were harvested from WT mice and stimulated *ex vivo* with 10 ng/ml PMA and 1 µg/ml ionamycin for 5 hours. (D) RNA was collected and evaluated by qRT-PCR for IL-6 expression. (E) Culture supernatants were harvested and evaluated for secreted IL-6 by electrochemilluminescence. Data were shown as the average IL-6 expression or protein ± SEM. A paired student's t-test was used to determine significance; *, *P*<0.05.

Previous studies using dextran sodium sulfate and T cell transfer models of colitis suggested monocytes and T cells are the source of IL-6 during these injuries [22], [23]. However, the data in our models demonstrated that IL-6 expression occurred quickly after injury, before monocytes and T cells have time to migrate into the injured tissue. Therefore, we sought to identify the initial source of IL-6 using *in situ* hybridization. In the endoscopic-guided biopsy injury model, *in situ* hybridization of IL-6 at 24 hours post-injury showed abundant IL-6 expressing cells located within highly proliferative crypts/wound channels that are located adjacent/within the wound bed (Figure 5A). Many of these cells were morphologically consistent with IELs based on size and position. These IL-6 expressing cells were not present in areas distant from the wound (Figure 5A). Similarly, by *in situ* hybridization in triggered *dnKO* mice, the majority of IL-6 positive cells at day six post-induction also appeared to be IELs (Figure 1A).

To determine if the IL-6 positive cells located within the colonic epithelium were IELs, we first tested if these cells were T cells. We performed immunofluorescence on colonic sections from *dnKO* mice six days post colitis induction using antibodies directed against IL-6 in combination with lineage markers. We found examples of cells within the epithelial layer with co-localization of CD3ε and IL-6 consistent with IEL lineage (Figure 5B). We next isolated IELs using established protocols (25) and found that, by flow cytometric analysis, >90% of CD3+ IL-6+ cells were TCRβ+ and CD4- CD8α- CD8β-. A lesser population, <10%, of CD3+ IL-6+ cells were TCRβ+ CD4+. Further, CD3+ IL-6+ cells were negative for TCRγδ and CD1d tetramer (Figure 5C and data not shown). To confirm that IELs can be induced to express IL-6, we used colonic IELs isolated from wild type mice for *in vitro* experiments. CD3+ CD4- CD8- epithelial lymphocytes stimulated with PMA for 5 hours expressed elevated levels of IL-6 mRNA and protein as compared to controls (Figure 5D, E). Taken together, these results support a role for IL-6 production in a population of IELs early after induction of injury.

### IL-6 expression is induced adjacent to acute perforations/injuries of the human intestine

**Figure 6:**
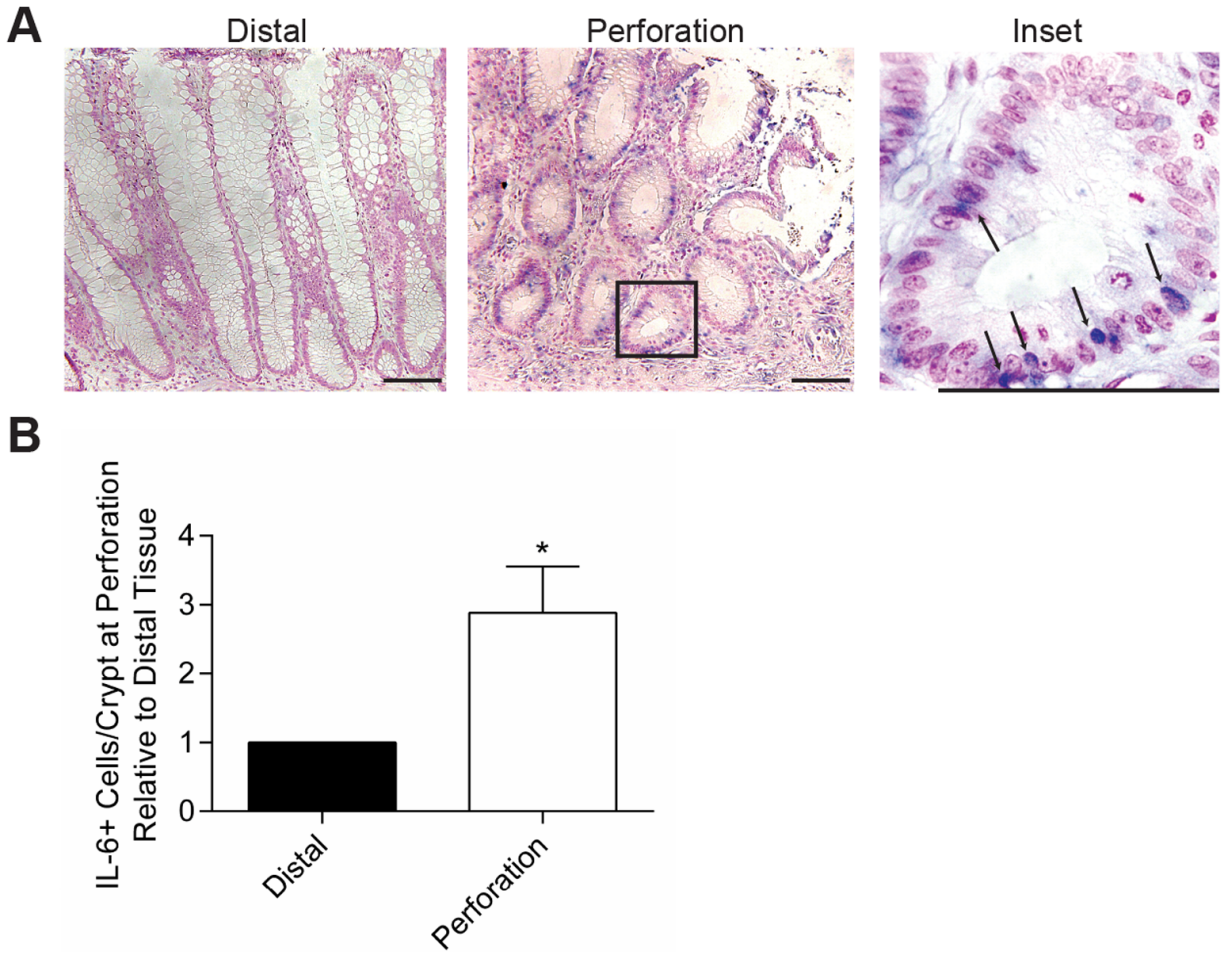
IL-6 expression was increased in human colons at sites of perforation. Tissue that was surgically resected from patients who suffered large bowel perforation was evaluated for IL-6 expression by *in situ* hybridization. Eleven cases were evaluated (8 males with trauma due to gun-shot wounds, ages 16–33; 1 female surgical trauma, age 45; and 2 females with diverticulitis, ages 72 and 79). (A) Representative staining from a patient with diverticulitis is shown at 20X and 100X, respectively. Bars = 100 µm. Arrows indicate IL-6+ cells with lymphocyte morphology. (B) Four high-powered fields with well-oriented crypts were evaluated for IL-6+ cells in the epithelial layer at the site of perforation and at the distal resection margin. The average ratio of IL-6+ cells in the perforation versus distal site ± SEM was shown. An unpaired student's t-test was used for statistical analysis; *, *P* = 0.02.

We performed a retrospective analysis of surgical bowel resection cases that were performed to excise areas of intestinal perforations that occurred due to a variety of etiologies (i.e. diverticulitis and trauma) to test if IL-6 expression was induced in areas of injury. For subsequent IL-6 expression analysis, we included only cases with histologic evidence of a perforation site in the intestine. For these cases, using adjacent unstained sections of the perforation site, we performed *in situ* hybridization to detect IL-6 mRNA. In all cases, IL-6 positive cells were readily detected at the injury site and the numbers of positive cells were enriched at the perforation sites as compared to sites distant from the perforation (Figure 6A, B). As anticipated, we detected IL-6 positive cells in the lamina propria. An unexpected result was that many cells with detectable IL-6 expression were localized within the epithelium and were morphologically consistent with intraepithelial lymphocytes (IELs, Figure 6A). The potential localization of IL-6 to IELs in areas of damage suggests a local impact of IL-6 on the intestinal epithelium. Of note, the epithelium near areas of perforation showed morphologic changes consistent with known alterations in mice that occur during repair. These changes included the formation of hypertrophic crypts adjacent to the injury that contained diminished cellular differentiation (i.e. goblet cells) and an expansion of proliferative progenitors.

## Discussion

In this report, we describe a novel role for IL-6 that supports intestinal epithelial repair and is dependent on timing. Early after bowel injury, we observe a substantial increase in IL-6 expression. The induction of IL-6 coincides with and is required for a burst of epithelial cellular proliferation that is known to be critical for epithelial repair. We found a functional role for IL-6 in intestinal proliferation in two different models of colonic injury, one inflammatory and the other physical. In mice, the induction of IL-6 occurs in IELs, which are interesting as they are in close contact with epithelial cells. The findings in mice appear to translate to human intestinal injuries as we can detect IL-6 expression in cells that are consistent with IELs by morphology and location.

Our finding that IL-6 stimulates epithelial proliferation is not unanticipated. In murine models of colon cancer, IL-6 increases the number and size of tumors, and IL-6 signaling through STAT3 promotes the growth and survival of tumor cells [13], [14], [22], [24]. Further, IL-6 is elevated in the sera of patients with colon cancer and has been suggested to promote tumor cell growth and survival [25]–[27]. The data we present here demonstrate that IL-6 stimulates epithelial proliferation during an acute injury for normal tissue repair in addition to chronic inflammation and tumorigenesis. Epithelial proliferation after injury is necessary for effective wound healing [21].

While IL-6 signaling in the epithelium appears to be beneficial during acute injury, its role during homeostasis is unclear. Unmanipulated *IL-6^-/-^* mice do not demonstrate an observable colon phenotype (data not shown, and reference [28]), and IL-6 expression was not detected in healthy colon tissues (Figures 2B and 3A), suggesting that this cytokine has no role in maintaining the epithelium during health. Further, in transgenic mice that overexpress IL-6, resulting in elevated levels of circulating IL-6, colon pathology has not been identified [29], [30]. Given our results, it is intriguing to consider local administration of IL-6 in the intestine after injury, such as diverticulitis, to promote healing.

Our studies focus on the role of IL-6 very early after injury. We identify IELs as a primary source of IL-6 in this early window, but do not discount the role of IL-6 produced by other cellular types and during later stages of disease. In dextran sodium sulfate induced colitis and the associated model of colitis-induced cancer, CD11b+ lamina propria mononuclear cells were the source for IL-6 that drove tumorigenesis several weeks after initiation of the disease model [22]. Transfer of CD4+ CD45RB^high^ T cells into severe combined immunodeficiency mice also results in colitis. In this model, IL-6 production at five weeks after cellular transfer was mainly derived from the transferred T cells. Transfer of T cells from IL-6 deficient mice resulted in reduced disease severity [23]. Finally, IL-6 has been identified in several cell types within tumors of mice with colitis-induced cancer including epithelial cells, CD4+ cells, and monocytes [24]. IL-6 production by sources other than IELs and at time points after the initial injury may explain our observation that blockade of IL-6 after colitis is induced in our spontaneous *dnKO* model did not result in improved disease. While we demonstrated a correction of the epithelial proliferation defect, mice treated with the inhibitory IL-6 antibody three days after induction of colitis still developed disease with increased crypt loss compared to control antibody treated *dnKO* mice. Thus, production of IL-6 from other cellular sources at later time points may lead to additional repair processes and/or tumorigenesis.

In patients with IBD, peripheral blood mononuclear cells and lamina propria mononuclear cells have been shown to secrete higher levels of IL-6 compared to cells from healthy control subjects. Yet, these results only correlated with active disease and were performed using patients with established disease [31], [32]. In a small, pilot clinical study, treatment of patients with active Crohn's disease with a humanized monoclonal antibody to IL-6R significantly improved disease activity scores but did not improve endoscopic and histologic scores [9]. These results suggest that IL-6 inhibition improves systemic inflammation but not wound healing of the intestine, which is in agreement with our data presented here. Therefore, further dissection of the role of IL-6 produced later in disease and by other cellular sources will be important if this pathway is to be a therapeutic target in IBD.

In conclusion, we demonstrate a beneficial role of IL-6 for intestinal wound healing early after injury. While inhibition of IL-6 proves successful for the treatment of systemic inflammatory disorders, much more needs to be understood as more specific targeting of the IL-6 pathway may reduce adverse events.

## Methods

### Animals and Housing

CRF2-4 (*IL-10R2^-/^* ^-^) and dominant negative *TGFβRII* mice on the C57BL/6 background were crossed to establish *dnKO* mice. Littermate *IL-10R2^+/−^* mice were used as controls for experiments. All mice were housed in a specific pathogen-free barrier facility in cages that were autoclaved after assembly and opened only in a laminar flow cabinet after disinfection with 1∶8∶1 dilution of Clidox-S (Pharmacal Research Laboratories, Inc., Naugatuck, CT). Cages were changed twice weekly. Mice were weaned at three weeks of age and placed on a solution of drinking water including 0.66 mg/ml ciprofloxacin (Sigma, St. Louis, MO), 2.5 mg/ml metronidazole (Sigma), and 20 mg/ml sugar-sweetened grape Kool-Aid mix (Kraft Foods, Deerfield, IL). Mice were treated with the antibiotic solution for three weeks prior to use in experiments. Colitis was induced by removing antibiotics from the drinking water and co-housing with untreated mice. Anti-IL-6 mAb treatment was initiated on day 0, at the time of co-housing, or on day 3 after co-housing. 500 µg of rat anti-IL-6 (clone MP5-20F3, provided by Pfizer, New York, NY) or rat anti-horseradish peroxidase as an isotype control antibody (Pfizer) was administered three times weekly by intraperitoneal injection.

*IL-6^-/-^* mice on the C57Bl/6 background were obtained from Jackson Laboratories (Bar Harbor, ME) and maintained as a colony in our mouse barrier facility.

All animal studies were performed under approval from the Washington University Animal Studies Committee.

### Histology

At the time of sacrifice mice were euthanized by cervical dislocation. Colons were harvested, flushed with PBS and methacarn, opened longitudinally, and pinned open with mucosa upward in square Petri dishes filled with wax (Carolina Biological, Burlington, NC). In this manner tissues were fixed in methacarn for four hours and then washed with 70% ethanol. Tissues were paraffin embedded and stained with hematoxylin and eosin. Representative images of histology were taken using an Olympus DP70 Digital Microscope Camera at 1360×1024 image size on an Olympus BX51 microscope.

### Immunofluorescence

For BrdU incorporation, mice were injected intraperitoneally with 150 µg BrdU (Sigma) per gram mouse weight one hour prior to sacrifice. Tissues were processed as above. 5 µm sections of paraffin embedded tissue were cut and fixed to glass slides. Slides were rehydrated by bathing 3 times each in xylenes for 3 minutes followed by isopropyl alcohol for 3 minutes. Slides were then rinsed in distilled water for 5 minutes. Antigen retrieval was performed with heated 10 mM sodium citrate buffer, pH 6.0. A hydrophobic pen was used to outline the tissue. Tissue was blocked with PBS containing 5% BSA and 0.05% Tween-20 for 10 minutes at room temperature. Goat anti-BrdU antibody (Abcam, Cambridge, MA) was diluted 1∶200 in blocking buffer and applied to the slides overnight at 4°C. After washing the slides in PBS for 5 minutes, secondary antibody donkey anti-goat IgG conjugated to 594 nm fluorochrome (Invitrogen, Grand Island, NY) diluted 1∶500 in blocking buffer was applied for 1 hour at room temperature. Again slides were washed for 5 minutes in PBS. Slides were then analyzed with a Zeiss Axiovert 200 microscope with an Axiocam MRM digital camera.

Co-localization of IL-6 positive cells was performed using frozen tissue sections. Mice were anesthetized by intraperitoneal injection of ketamine 87 mg/kg and xyloxine 13 mg/kg mouse bodyweight. The mice were perfused with 4% paraformaldehyde in PBS by intracardiac injection. Colons were harvested, flushed with PBS, and fixed in 4% paraformaldehyde overnight. After washing the tissue with 20% sucrose, it was embedded in OCT. 5 µm tissue sections were cut and fixed to glass slides with cold acetone for 10 minutes. Tissue was outlined with a hydrophobic pen and blocked with PBS containing 5% BSA and 0.05% Tween-20 for 10 minutes at room temperature. Rat anti-IL-6 (clone MP5-20F3, provided by Pfizer) diluted in blocking buffer to a concentration of 50 µg/ml was applied to the tissue for one hour at room temperature. After washing tissue in PBS for 5 minutes, the secondary antibody anti-rat IgG 594 nm fluorochrome-conjugated (Invitrogen) diluted 1∶500 in blocking buffer was applied for one hour at room temperature. Again the tissue was washed for 5 minutes in PBS. FITC-conjugated rat anti-CD3ε (clone 145-2C11, BD Biosciences, San Jose, CA) diluted 1∶50 in blocking buffer was applied for one hour at room temperature. Slides were washed for 5 minutes in PBS, nuclei stained with bis-benzimide, washed, and coverslips applied with glycerol-PBS. Tissue staining was evaluated with a Zeiss Axiovert 200 microscope with an Axiocam MRM digital camera.

### In situ Hybridization

IL-6 RNA probe was established by reverse transcription of cDNA (for mouse IL-6: IMAGE ID 8861788, Source BioScience, Nottingham, United Kingdom; for human IL-6: IMAGE ID 3884652, Thermo Scientific. Waltham, MA) using digoxigenin-labeled ribonucleotides (Roche, Indianapolis, IN). Human tissue was obtained from banked pathology specimens under approval from Washington University Institutional Review Board. Sections of paraffin-embedded human tissue were rehydrated in xylenes followed by a series of alcohols. Fixed frozen mouse tissue prepared as above was fixed to glass slides with cold 4% paraformaldehyde in PBS. Tissue was then digested with 10 ng/ml proteinase K followed by treatment with acetic anhydride. The probe was allowed to hybridize overnight at 60°C in chambers moisturized with 50% formamide. Following hybridization, RNA was digested with 1 µg/ml RnaseA. RNA labeling was then detected using an alkaline phosphatase-conjugated anti- digoxigenin antibody (Roche) diluted 1∶2000. Intrinsic alkaline phosphatases were inactivated by incubation with 5 mM levamisole. Staining was detected with either Alkaline Phosphatase Kit I (Vector Laboratories, Burlingame, CA) or 75 µg/ml NBT combined with 175 µg/ml BCIP (Roche).

### qRT-PCR

Cells in culture were harvested and RNA isolated using the NucleoSpin RNA II kit (Macherey-Nagel, Bethlehem, PA). cDNA was created with Superscript III reverse transcriptase (Life Technologies, Carlsbad, CA). Reactions consisted of 1 µl cDNA, 0.6 µM each forward and reverse primers, 1x SYBR Green (Clontech Laboratories, Mountain View, CA), and water for a total volume of 17 µl. Samples were denatured at 95°C for 2 minutes, cycled 40 times through 95°C for 20 seconds, 58°C for 20 seconds, and 72°C for 30 seconds, and then denaturation curves determined from 58°C through 95°C. Primer sequences were as follows: glyceraldehyde 3-phosphate dehydrogenase forward, 5′-AGGTCGGTGTGAACGGATTTG-3′; glyceraldehyde 3-phosphate dehydrogenase reverse, 5′-TGTAGACCATGTAGTTGAGGTCA-3′; IL-6 forward, 5′-CTCTGC AAGAGACTTCCATCCAGT-3′; and IL-6 reverse 5′-GAAGTAGGGAAGGCCGTGG-3′. All qPCR assays were conducted in a Mastercycler ep realplex real-time PCR machine (Eppendorf, Hamburg, Germany). Specificity of amplicon was verified by agarose gel electrophoresis.

### IL-6 ELISA

Sera were collected from mice via retro-orbital bleed. Sera were assayed for IL-6 by a commercially available ELISA (Mouse IL-6 ELISA MAX, Biolegend, San Diego, CA). IL-6 in IEL culture supernatant was evaluated by electrochemilluminescence using a commercially available kit and detected with a Sector Imager 2400 (Meso Scale Discovery, Rockville, MD).

### Flow Cytometry

Epithelial cells from the colons of mice were harvested as described [33]. Isolated, enriched IELs were stained for flow cytometry by incubating cells with antibodies to the following cell-surface antigens: CD3ε, CD4, CD8α, CD8β, TCRβ, (eBioscience, San Diego, CA) TCRγδ, and CD1d tetramer loaded with α-Gal-Cer peptide and unloaded control tetramer (NIH Tetramer Facility). Intracellular staining for IL-6 was done after surface staining and fixing cells (Cytofix/Cytoperm Fixation/Permeabilization Solution Kit, BD Biosciences). Stained cells were analyzed using BD LSR Fortessa and FloJo X software.

### IEL Isolation and Culture

Colons were removed from mice, flushed with cold PBS, and hemisected longitudinally. Tissue was then placed in Hanks' balanced salt solution without Ca^2+^ and Mg^2+^ with the addition of 10% FCS and 1 mM EDTA. Tissue was vortexed for 10 minutes at room temperature. After pouring through a cell strainer, cells were pelleted by centrifugation. CD3+ CD4- CD8- cells were negatively selected by magnetic sorting using antibodies to CD4, CD8α, EpCAM (0.5 µg each) and EasySep Mouse T Cell Isolation Kit (Stem Cell Technologies, Vancouver, BC, Canada). Isolated cells were placed in culture with RPMI 1640 supplemented with 10% FCS, 100 U/ml penicillin, 100 µg/ml streptomycin, and 2 mM L-glutamine. 10 ng/ml PMA and 1 µg/ml ionamycin were added to cultured cells for 5 hours before harvesting supernatants and RNA.

## Supporting Information

Figure S1 **Anti-IL-6 treatment reduced serum IL-6 levels.** Colitis was induced in *dnKO* and *IL-10rb^+/−^* littermate controls. At day 0 and three times weekly, mice were treated with 500 µg anti-IL-6 antibody or control IgG1. Sera were collected at the time of sacrifice (day 9 after induction of colitis), and IL-6 was measured by ELISA. Data are the mean ± SEM for each treatment group.
(TIF)

Figure S2 **IL-6 was necessary for proliferation of epithelial cells in wound channels.** Endoscopic-guided biopsy was performed on *IL-6^-/-^* and *IL-6^+/−^* littermate control mice. Immunofluorescence of wounds from day 6 after injury was performed for Ki67 (proliferating cells, pink), β-catenin (epithelium, green), and bis-benzimide (nuclei, blue). Representative staining is shown at 10X. Bars = 500 µm.
(TIF)
