# Supplementary figures and images for: IL-6 Stimulates Intestinal Epithelial Proliferation and Repair after Injury
Source: PLoS One. 2014 Dec 5;9(12):e114195. doi: 10.1371/journal.pone.0114195 (PMC4257684; doi:10.1371/journal.pone.0114195)

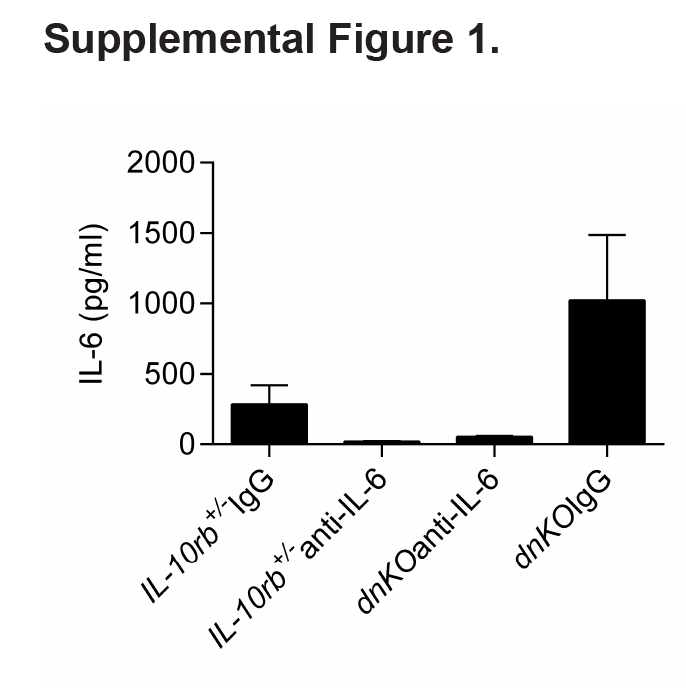

Supplement: Figure S1 — Anti-IL-6 treatment reduced serum IL-6 levels. Colitis was induced in dnKO and IL-10rb+/− littermate controls. At day 0 and three times weekly, mice were treated with 500 µg anti-IL-6 antibody or control IgG1. Sera were collected at the time of sacrifice (day 9 after induction of colitis), and IL-6 was measured by ELISA. Data are the mean ± SEM for each treatment group. (TIF) [file pone.0114195.s001.tif]

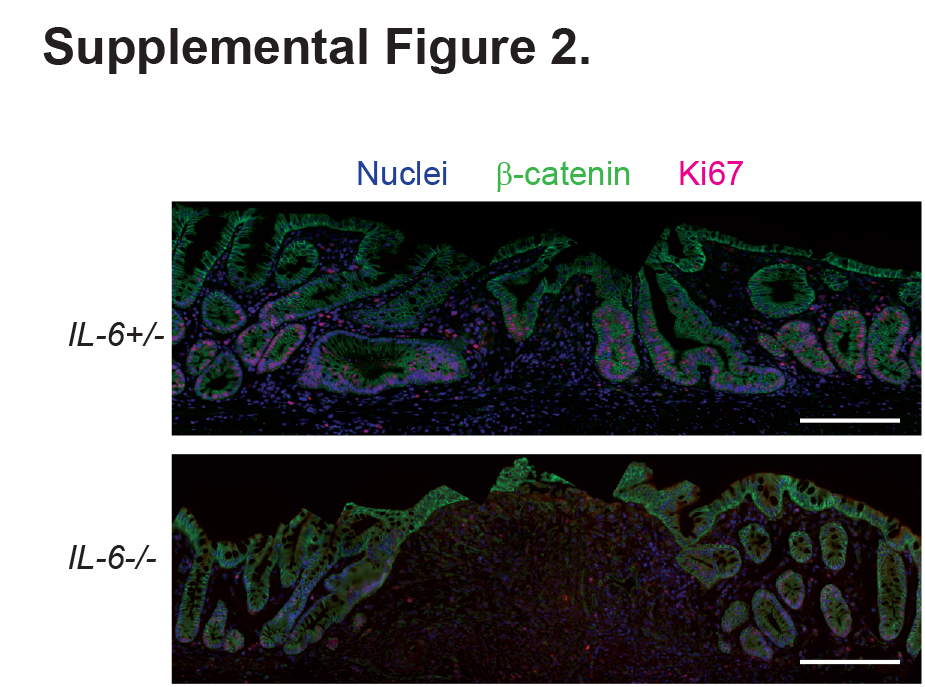

Supplement: Figure S2 — IL-6 was necessary for proliferation of epithelial cells in wound channels. Endoscopic-guided biopsy was performed on IL-6-/- and IL-6+/− littermate control mice. Immunofluorescence of wounds from day 6 after injury was performed for Ki67 (proliferating cells, pink), β-catenin (epithelium, green), and bis-benzimide (nuclei, blue). Representative staining is shown at 10X. Bars = 500 µm. (TIF) [file pone.0114195.s002.tif]
